# Contraceptive use and the intention to become pregnant among women attending the Brazilian Unified Health System

**DOI:** 10.1590/1518-8345.3451.3328

**Published:** 2020-08-12

**Authors:** Celia Regina Maganha e Melo, Ana Luiza Vilela Borges, Luciane Simões Duarte, Natália de Castro Nascimento

**Affiliations:** 1 Universidade de São Paulo, Escola de Artes, Ciências e Humanidades, São Paulo, SP, Brazil.; 2 Universidade de São Paulo, Escola de Enfermagem, São Paulo, SP, Brazil.

**Keywords:** Contraception, Sexual and Reproductive Health, Intention, Women’s Health, Primary Health Care, Nursing, Anticoncepção, Saúde Sexual e Reprodutiva, Intenção, Saúde da Mulher, Atenção Primária à Saúde, Enfermagem, Anticoncepción, Salud Sexual y Reproductiva, Intención, Salud de la Mujer, Atención Primaria de Salud, Enfermería

## Abstract

**Objective:**

to analyze the use of contraceptive methods and the intention to become pregnant among women attending the Brazilian Unified Health System.

**Method:**

a cross-sectional study conducted with 688 women aged 18-49 years old, attending the Family Health Strategy Facilities in the eastern part of the city of São Paulo, Brazil, who were awaiting medical or nursing consultation. Data were obtained through interviews with a structured instrument, allocated in tablets. The analysis was conducted with “strong desire to avoid pregnancy” as the dependent variable. Chi-square and multiple logistic regression were used, calculated in Stata 14.2.

**Results:**

56.5% used some contraceptive method, covariates of the strong desire to avoid pregnancy were marital status (OR=0.49; CI95%=0.33-0.74), parity – two and more children (OR=15.9; IC95%=4.29-59.1); and pregnancy planning – planned (OR=0.69; IC95%=0.73-0.94) and ambivalent (OR=2.94; IC95%=1.30-3.83). There was no statistical difference between the strong desire to avoid pregnancy and the type of contraceptive used.

**Conclusion:**

women with a strong desire to avoid pregnancy used basically the same types of contraceptive methods as women in general, which shows that they have not been supported to achieve their reproductive preferences.

## Abstract (pt)

**Objetivo:**

analisar o uso de métodos contraceptivos e intencionalidade de engravidar entre mulheres atendidas no Sistema Único de Saúde.

**Método:**

estudo transversal conduzido com 688 mulheres de 18-49 anos de idade, usuárias de Unidades Estratégia Saúde da Família da zona Leste da cidade de São Paulo, Brasil, que aguardavam consulta médica ou de enfermagem. Os dados foram obtidos por meio de entrevista com instrumento estruturado, alocado em *tablets*. A análise foi conduzida tendo como variável dependente “forte desejo de evitar a gravidez”. Utilizou-se qui-quadrado e regressão logística múltipla, calculados no Stata 14.2.

**Resultados:**

56,5% usaram algum método contraceptivo; covariáveis do forte desejo de evitar a gravidez: estado civil (OR= 0,49; IC 95% = 0,33-0,74), paridade - dois e mais filhos (OR = 15,9; IC95% = 4,29- 59,1); e planejamento da gravidez - planejado (OR = 0,69; IC95% = 0,73-0,94) e ambivalente (OR = 2,94; IC95% = 1,30-3,83). Não houve diferença estatística entre o forte desejo de evitar a gravidez e o tipo de contraceptivo utilizado.

**Conclusão:**

As mulheres com forte desejo de evitar a gravidez usavam basicamente os mesmos tipos de métodos contraceptivos que as mulheres em geral, o que mostra que elas não foram apoiadas para alcançar suas preferências reprodutivas.

## Abstract (es)

**Objetivo:**

analizar el uso de métodos anticonceptivos y la intencionalidad de embarazo entre las mujeres que usan el Sistema Público de Salud Brasileño, SUS.

**Método:**

estudio transversal realizado con 688 mujeres de entre 18 y 49 años, usuarias de las Unidades de Estrategia de Salud Familiar en la zona este de la ciudad de São Paulo, Brasil, que esperaban por su cita médica o de enfermería. Datos obtenidos a través de entrevistas con un instrumento estructurado, cargado en *tablets*. El análisis se realizó con el “fuerte deseo de evitar el embarazo” como variable dependiente. Se utilizó regresión logística múltiple y prueba de chi-cuadrado, calculadas en Stata 14.2.

**Resultados:**

56,5% utilizaba algún método anticonceptivo; 56.5% utilizó algún método anticonceptivo, covariables del fuerte deseo de evitar el embarazo: estado civil (OR= 0.49; IC 95% = 0.33-0.74), paridad - dos y más niños (OR = 15 , 9; IC 95% = 4.29-59.1); y planificación del embarazo: planeado (OR = 0,69; IC 95% = 0,73-0,94) y ambivalente (OR = 2,94; IC 95% = 1,30-3,83). No hubo diferencia estadística entre el fuerte deseo de evitar el embarazo y el tipo de anticonceptivo utilizado.

**Conclusión:**

las mujeres con un fuerte deseo de evitar el embarazo utilizaron básicamente los mismos tipos de métodos anticonceptivos que las mujeres en general, lo que demuestra que no recibieron apoyo para lograr sus preferencias reproductivas.

## Introduction

Sexual health is widely understood as a state of physical, emotional, mental, and social well-being in relation to sexuality. It is not only related to certain aspects of reproductive health, but also to the possibility of having a pleasant and safe sexual life, free from coercion, discrimination, and violence. Achieving the highest attainable standard of sexual health is closely linked to the respect, protection, and fulfillment of human rights, non-discrimination, privacy and confidentiality, to be free from violence and coercion, as well as the rights to education, information, and access to the health services^(^ 1 ^)^.

In the Action Program of the 1994 International Conference on Population and Development^(^ 2 ^-^ 3 ^)^, the governments committed to enabling people to make choices about their sexual and reproductive health considering the fundamental human rights, as millions of women globally want to avoid pregnancy, but neither they nor their partners use a contraceptive, or use them inappropriately and discontinuously, or even use ineffective and short duration methods^(^ 4 ^)^.

On the other hand, many women worldwide use contraceptive methods to prevent pregnancy, but they fail for a number of reasons, such as not having received clarifying instructions on how to use the method properly, not having obtained the method best suited to their clinical, social, and reproductive health needs, and limited availability of health services^(^ 5 ^)^.

Brazil has shown a significant drop in the fertility rate in the last decades, from 6.3 children *per* woman in 1960 to 1.7 children in 2018^(^ 6 ^)^. The historical series of the Demographic and Health Survey (*Pesquisa Nacional sobre Demografia e Saúde*, PNDS) show that the contraceptive prevalence rate among married women aged 15 to 49 years old increased from 66.2% in 1986 to 80.6% in 2006^(^ 7 ^)^.

The occurrence of unintended pregnancies represented 44% of the pregnancies that occurred in the five years prior to PNDS 2006^(^ 8 ^)^, findings ratified by the *Nascer* no *Brasil* (Being born in Brazil) survey of 2014, which indicates that 55.4% of the pregnancies were unintended. It is known that unintended pregnancies can have negative effect on women and their children; they contribute to the occurrence of induced abortions resulting in the main cause of maternal mortality in countries with restrictive abortion laws. In addition, unintended births are associated with an increased risk of obstetric complications, late prenatal care and babies more likely to have low birth weight, premature birth, and maternal depression^(^ 9 ^-^ 10 ^)^.

A study conducted in Oklahoma, USA, examined the effect of pregnancy intentions at three different times: the prenatal period, the immediate postpartum period, and the period of early childhood. The estimated effects were stronger in the prenatal period and decreased at two years of age, suggesting that, over time, mothers adjust to unintended births and respond to the health needs of their young children, regardless of the status of the pregnancy intention. In the prenatal period, women with unintended pregnancies were less likely to engage in health-promoting behaviors than women with intended pregnancies^(^ 11 ^)^.

That said, it is essential to assess women’s access to family planning and track their reproductive intentions and preferences, precisely to support the provision of counseling and contraceptive supplies according to their needs and preferences. Regarding the responsibilities that the public health system has in guaranteeing the implementation of women’s sexual and reproductive rights, the Family Health Strategy (FHS) (*Estratégia Saúde da Família*, ESF) plays an important role. For the proper functioning of the program, it is essential that the primary health care facility make the various contraceptive methods available and, in an amount compatible with the local reality, because the lack of supplies limits the choice of users and imposes the use of a certain method without observing individual needs. The guarantee of sufficient contraceptives for users ensures equal access to methods and their absence constitutes a denial of a constitutional right^(^ 12 ^)^.

Thus, it is assumed that the context of inadequate family planning services can cause discrepancies between the reproductive preferences of women attending the Unified Health System (*Sistema* Único *de Saúde*, SUS) and their contraceptive practices, i.e., it is not known to what extent the contraceptive practices of women attending the SUS are subsidized to decide about contraception based on an individual and informed choice or are due to the limitations of family planning services, which leads them to use contraceptive methods that are little or nothing congruent with their reproductive preferences and intention to becoming pregnant.

Therefore, the aim of this study is to analyze the use of contraceptive methods and the intention to become pregnant among women attending the SUS, as well as to describe the contraceptive methods used by them according to their reproductive intention. Our findings may facilitate women’s decision-making by establishing public policies investment priorities, aiming to meet the reproductive intention and the services offered in Primary Health Care.

## Method

This is a quantitative and cross-sectional study carried out in the East part of the city of São Paulo, Brazil. The study population consisted of a probabilistic sample of women aged 18 to 49 years old, attending the Family Health Strategy in the East part of the city of São Paulo, specifically from the micro-region of Itaim Paulista.

The sample size calculation was based on the probability sampling technique^(^ 13 ^)^, with which, from a sample, one can generalize the characteristics of the population and expand the data to the group of women between 18 and 49 years old from the city of São Paulo. In view of the purpose of this study, for dimensioning the sample size (n), we chose to use the “use of contraceptive methods” variable as a parameter, in order to estimate the percentage of women aged 18 to 49 years old who have already used contraceptive methods at least once in their lifetime in the Southeastern region, according to PNDS 2006^(^ 14 ^)^. The calculation showed that it would be necessary to interview 684 women in the established age group. Four Primary Health Care Facilities, out of ten Family Health Strategy units in the micro-region of Itaim, were randomly selected, and women awaiting medical or nursing consultation at the FHS facilities were invited to participate in the study. The inclusion criterion was having started sexual life; and the exclusion ones were being current pregnant, having tubal ligation, and having a vasectomized partner. The number of women interviewed in each health facility was 171. For those eligible, the research objectives were explained and, after acceptance, Informed Consent Term was read and signed.

To carry out data collection, a team of trained female researchers, undergraduates, and health professionals was formed with previous experience in data collection with face-to-face interviews. The field researchers were continuously supervised by the research coordinators, through follow-up visits in the Family Health Strategy facilities and during meetings to deliver interviews held during the week.

The structured instrument used for data collection consisted of questions about sociodemographic characteristics, reproductive history, use of contraceptive methods, and reproductive intention.

The data were collected on tablets and managed using the electronic data capture tools of the Research Electronic Data Capture (REDCap)^(^ 15 ^)^. Field work took place from December 2017 to February 2018, during working days of the week, in the morning and afternoon. Data analysis was performed using the Stata software, version 14.0, divided into the following stages: a) characterization of the sociodemographic profile, reproductive and contraceptive behavior of women, in addition to reproductive intention, using numbers and proportions. Sociodemographic covariates were age (18-24; 25-34; 35 and over); self-declared race/skin color (white, brown, black, yellow); religion (none, catholic, protestant, others); schooling (up to 8 years, 9 years or more); marital status (has a partner: yes, no); own source of income (yes or no); health insurance (yes or no); and socioeconomic status (A/BC/D/E, according to the Brazil Economic Classification Criterion 2015^(^ 16 ^)^). For the analysis of the reproductive and contraceptive behavior, the following variables were analyzed: age at menarche; age of sexual initiation; number of sexual partners in life; previous pregnancy (yes or no); age at first pregnancy; number of pregnancies; history of abortion (yes or no), and number of children.

In order to analyze the relation between reproductive preferences and contraceptive practices, the measurement of pregnancy planning using the London Measure of Unplanned Pregnancy (LMUP), Brazilian version, was chosen. This instrument is short, and consists of six items that make up the pregnancy-planning domain. One of the potentialities of the LMUP is the classification beyond the dichotomous and artificial position of “planned” or “did not plan the pregnancy”, since it makes it possible to classify women as having an ambivalent pregnancy planning. This means that the instrument does not ignore the complexity of female experiences related to reproduction, including ambivalences or uncertainties^(^ 17 ^)^. The use of contraceptives was defined as the use of any method during the period of interview. The variable “strong desire to avoid pregnancy” was created by combining the variables “I would like to get pregnant” (do not want to get pregnant (any more), or immediately, between 1 and 2 years, 2 years or more, don’t know/not sure); “Importance of preventing pregnancy” (very important, indifferent, not very important); “Time to become pregnant” (wrong, neither right/nor wrong, right); and “Feelings if an unexpected pregnancy occurs” (sad/unhappy, indifferent, don’t know, happy). Therefore, the “strong desire to avoid pregnancy” variable is the sum of the codes of these four variables, which varied from 4 to 12: the higher the score, the stronger the desire to avoid pregnancy; and the lower the score, the weaker the desire to avoid pregnancy. The variable, however, was analyzed in a dichotomous way, with code 0 (zero) being attributed to women whose score was up to 10 and code 1 (one) to women whose score were 11 and 12, i.e., they reported at least three situations that express they really did not want to get pregnant, among the four possible^(^ 18 ^)^.

The aspects associated with having a strong desire to avoid pregnant were analyzed using multiple logistic regression, in which the variables were inserted simultaneously in the model. The main independent variable was the type of contraceptive method used. This variable considered the effectiveness of the reversible and permanent methods according to the Effectiveness of Family Planning Methods^(^ 19 ^)^ being “High efficacy” (less than 01 pregnancies *per* 100 women/year); “Medium efficacy” (6-12 pregnancies per 100 women/year) and “Low efficacy” (18 or more pregnancies per 100 women/year).

The study followed the ethical precepts of Resolution 466/2012 and was approved by the Research Ethics Committee with Opinion nº 60967616.5.0000.5390.

## Results

We approached 847 women who were awaiting medical or nursing consultation, in which 688 were eligible, 72 refused to participate and 87 were ineligible. Among the 688 women who were interviewed, 255 (37.7%) were between 25 and 34 years old, 573 (83.3%) attended high school, 308 (44.8%) practiced the Evangelical religion, 508 (73.9%) declared themselves to be non-white, 444 (64.5%) did not have a partner, 365 (53.1%) did not have their own income, 565 (82.1%) had no health insurance, and 479 (70.9%) fell into socioeconomic status C. As for parity, 297 (43.2%) had two or more children, 266 (62.1%) revealed that they would not like to have (more) children, and 389 (56.5%) were using some contraceptive method; 179 (41.8%) women would not like to have (more) children, being associated with age (p<0.001) and parity (p<0.001). The use of contraceptive methods (CCMs) was not associated with the number of women who would not like to have (more) children, age, and parity (Table 1).

**Table 1:** Characteristics and proportion of the women who would or would not like to have (more) children according to socioeconomic and demographic variables. São Paulo, SP, Brazil, 2018

Variable	I would like to have (more) children							
	N	%	No	%	Yes	%	Does not know	%
Age (years old)				p<0.001				
18-24	214	31.1	86	20.1	111	50.2	17	43.6
25-34	255	37.7	162	37.8	79	35.7	14	35.9
35+	219	31.9	180	42.1	31	14.0	8	20.5
Schooling				p 0.061				
Elementary	21	3.0	15	3.5	5	2.3	1	2.6
High School	573	83.3	367	85.7	174	78.7	32	82.0
Higher	94	13.7	46	10.7	42	19.0	6	15.4
Religion				p 0.121				
None	176	26.4	113	26.4	59	26.7	4	10.3
Catholic	175	25.4	117	27.3	49	22.2	9	23.1
Evangelical	308	44.8	179	41.8	104	47.1	25	64.1
Others	29	4.22	19	4.4	9	4.1	1	2.6
Declared skin color*				p 0.094				
White	179	26.1	102	23.9	62	28.2	15	38.5
Non-white	508	73.9	326	76.2	158	71.8	24	61.5
Has a partner				p 0.412				
Yes	244	35.5	144	33.6	84	38.0	16	41.0
No	444	64.5	284	66.4	137	62.0	23	59.0
Own income				p 0.367				
No	365	53.1	225	52.6	115	52.3	25	64.1
Yes	322	46.9	203	47.4	105	47.7	14	35.9
Health insurance				p 0.006				
No	565	82.1	363	84.8	167	75.6	35	89.7
Yes	123	17.9	65	15.2	54	24.4	4	10.3
Socioeconomic status*								
A+B	158	23.4	92	21.7	55	2536	11	28.9
C	479	70.9	303	71.6	151	70.2	25	65.8
D+E	39	5.8	28	6.6	9	4.2	2	5.3
Parity				p<0.001				
None	128	18.6	33	7.7	82	37.1	13	33.3
1 child	263	38.2	129	30.1	114	51.6	20	51.3
2+ children	297	43.2	266	62.1	25	11.3	6	15.4
Uses CMs^†^				p 0.422				
No	299	43.5	179	41.8	104	47.1	16	41.0
Yes	389	56.5	249	58.2	117	53.0	23	59.0
Total	688	100	428	100	221	100	39	100

* Some women did not answer;

† CMs = Contraceptive methods

In order to know the intention of getting pregnant, women were asked whether or not they would like to have (more) children, and the results were statistically significant for age (p<0.001), no health insurance (p<0.005), and parity (p<0.001). Regarding how important it was to prevent pregnancy, there was an association between parity (p<0.001) and not using any contraception method (p<0.001), according to Table 2.

**Table 2:** Number and proportion of women according to intent and importance in preventing pregnancy. São Paulo, SP, Brazil, 2018

Variable	Wants to have (more) children			Important to prevent pregnancy		
	No	Yes	Does not know	Very much	Indiff.*	Little
	n(%)	n(%)	n(%)	n(%)	n(%)	n(%)
Age (years old)		p <0.001			p 0.282	
18-24	98(21.7)	113(51.6)	3(16.7)	195(32.4)	7(20.6)	12(23.1)
25-34	169(37.4)	75(34.2)	11(61.1)	223(37.0)	13(38.2)	19(36.5)
35 and over	184(40.8)	31(14.2)	4(22.2)	184(30.6)	14(41.2)	21(40.4)
Schooling		p 0.063			p 0.168	
Elementary	15(3.3)	5(2.3)	1(5.6)	17(2.8)	1(2.9)	3(5.8)
High School	386(85.6)	172(78.5)	15(83.3)	506(84.0)	24(70.6)	43(82.7)
Higher	50(11.1)	42(19.2)	2(11.1)	79(13.1)	9(26.5)	6(11.5)
Socioeconomic status		p 0.735			p 0.980	
A and B	98(22.0)	56(26.4)	4(22.2)	139(23.4)	9(26.5)	10(20.4)
C	320(71.7)	146(69.0)	13(72.2)	420(70.8)	23(67.6)	36(73.5)
D and E	28(6.3)	10(4.7)	1(11.1)	34(5.7)	2(5.9)	3(6.1)
Declared skin color		p 0.208			p 0.302	
White	108(23.9)	65(29.8)	6(33.3)	151(25.0)	11(33.3)	17(32.7)
Non-white	343(76.0)	153(70.2)	12(66.7)	451(74.9)	22(66.8)	35(67.3)
Religion		p 0.557			p 0.444	
None	118(26.1)	55(25.1)	3(16.7)	158(26.2)	9(26.5)	9(17.3)
Catholic	122(27.0)	48(21.9)	5(27.8)	149(24.7)	8(23.5)	18(34.6)
Evangelical	191(42.3)	107(48.9)	10(55.6)	272(45.2)	14(41.2)	22(42.3)
Other	20(4.4)	9(4.1)	-	23(3.8)	3(8.8)	3(5.8)
Own income		p 0.507			p 0.894	
No	238(52.7)	115(52.7)	12(66.7)	321(53.4)	18(52.9)	26(50.0)
Yes	213(47.2)	103(47.2)	6(33.3)	280(46.6)	16(47.1)	26(50.0)
Health insurance		p 0.005			p 0.886	
No	386(85.6)	165(75.3)	14(77.8)	493(81.9)	28(82.3)	44(84.6)
Yes	65(14.4)	54(24.7)	4(22.2)	109(18.1)	6(17.6)	8(15.4)
Stable union		p 0.312			p 0.761	
No	156(34.6)	84(38.4)	4(22.2)	216(35.9)	12(35.3)	16(30.8)
Yes	295(65.4)	135(61.6)	14(77.8)	386(64.1)	22(64.7)	36(69.2)
Parity (children)		p <0.001			p <0.001	
0	40(8.9)	88(40.2)	-	98(16.3)	14(41.2)	16(30.8)
1	142(31.5)	108(49.3)	13(72.2)	228(37.9)	12(35.3)	23(44.2)
2 and over	269(59.6)	23(10.5)	5(27.8)	276(45.9)	8(23.5)	13(25.0)
LMUP^†^		p 0.182			p 0.107	
Planned	142(34.9)	52(37.4)	8(44.4)	174(34.5)	11(52.4)	17(43.6)
Ambivalent	181(44.5)	69(49.6)	9(50.0)	231(45.8)	9(42.9)	19(48.7)
Not planned	84(20.6)	18(12.9)	1(5.6)	99(19.6)	1(4.8)	3(7.7)
Uses CMs^‡^		p 0.021			p <0.001	
No	190(42.1)	106(48.4)	3(16.7)	237(39.4)	23(67.6)	39(75.0)
Yes	261(57.9)	113(51.6)	15(83.3)	365(60.6)	11(32.4)	13(25.0)
Total	451(100)	219(100)	18(100)	602(100)	34(100)	52(100)

* Indiff. = Indifferent;

† LMUP = London Measure of Unplanned Pregnancy;

‡ CMs = Contraceptive methods

Women were asked how they would consider that moment if they got pregnant. The results were statistically significant for parity (p<0.001) and the use of contraceptive method (p<0.001). Regarding the feeling related to an unexpected pregnancy, there was an association between parity (p<0.001) and pregnancy planning (p<0.001) (Table 3).

**Table 3:** Number and proportion of women according to how they consider the time of a pregnancy and the feeling regarding an unplanned pregnancy. São Paulo, SP, Brazil, 2018

Variable	Time of pregnancy			Feeling towards unexpected pregnancy			
	Wrong	Does not know	Right	Sad	Indiff.*	Does not know^†^	Happy
	n(%)	n(%)	n(%)	n(%)	n(%)	n(%)	n(%)
Age (years old)		p 0.219			p 0.504		
18-24	141(32.9)	44(33.8)	29(22.5)	57(28.0)	15(32.6)	56(36.1)	86(30.4)
25-34	153(35.7)	48(37.0)	54(41.9)	85(41.7)	15(32.6)	48(31.0)	107(38.0)
35+	135(31.5)	38(29.2)	46(35.7)	62(30.4)	16(34.8)	51(34.8)	90(31.8)
Schooling		p 0.308			p 0.144		
Elemen.^‡^	16(3.7)	1(0.8)	4(3.1)	11(5.4)	- (0.0)	4(2.6)	6(2.1)
High School	360(83.9)	110(84.6)	103(79.8)	166(81.4)	41(89.1)	135(87.1)	231(81.7)
Higher	53(12.3)	19(14.6)	22(17.0)	27(13.2)	5(10.9)	16(10.3)	46(16.2)
Socioeconomic group^§^		p 0.581			p 0.411		
A+B	94(22.2)	33(25.8)	31(25.0)	45(22.4)	14(31.1)	30(19.7)	69(24.8)
C	304(71.7)	86(67.2)	89(71.8)	144(71.6)	30(66.7)	109(71.7)	196(70.5)
D+E	26(6.1)	9(7.0)	4(3.2)	12(6.0)	1(2.2)	13(8.5)	13(4.7)
Skin color		p 0.514			p 0.413		
White	106(24.7)	35(26.9)	38(29.7)	49(24.0)	11(23.9)	36(23.2)	83(29.4)
Non-white^‖^	323(75.3)	95(73.1)	90(70.3)	155(76.0)	35(76.1)	119(76.8)	199(70.6)
Religion		p 0.947			p 0.500		
None	110(25.6)	34(26.1)	32(24.8)	53(26.0)	12(26.1)	45(29.0)	66(23.3)
Catholic	110(25.6)	36(27.7)	29(22.5)	47(23.0)	13(28.3)	43(27.7)	72(25.4)
Evangelical	190(44.3)	56(43.1)	62(48.1)	98(48.0)	20(43.5)	57(36.8)	133(47.0)
Other	19(4.4)	4(3.1)	6(4.6)	6(2.9)	1(2.2)	10(6.4)	12(4.2)
Own income^§^		p 0.922			p 0.424		
No	229(53.5)	67(51.5)	69(53.5)	103(50.5)	22(49.0)	79(51.0)	161(57.0)
Yes	199(46.5)	63(48.5)	60(46.5)	101(49.5)	23(51.0)	76(49.0)	122(43.0)
Health insurance		p 0.749			p 0.575		
No	356(83.0)	105(80.8)	104(80.6)	166(81.4)	38(82.6)	133(85.8)	
Yes	73(17.0)	25(19.2)	25(19.4)	38(18.6)	8(17.4)	22(14.2)	
Has a partner		p 0.007			p 0.249		
No	171(39.9)	39(30.0)	34(26.4)	77(37.7)	18(39.1)	61(39.3)	88(31.1)
Yes	258(60.1)	91(70.0)	95(73.6)	127(62.2)	28(60.9)	94(60.6)	195(69.0)
Parity		p<0.001			p<0.001		
None	59(13.7)	26(20.0)	43(33.3)	15(7.3)	9(19.6)	26(16.8)	78(27.6)
01 child	160(37.3)	54(41.5)	49(38.0)	64(41.3)	19(41.3)	64(41.3)	113(40.0)
02+	210(49.0)	50(38.5)	37(28.7)	122(59.8)	18(39.1)	65(41.9)	92(32.5)
LMUP^§^		p 0.053			p<0.001		
Planned	123(33.5)	40(38.1)	39(42.4)	51(27.4)	17(43.6)	38(29.2)	96(46.9)
Ambiv.^‖^	167(45.5)	55(52.4)	37(40.2)	91(48.9)	10(25.6)	66(50.8)	92(44.0)
N. pl.^¶^	77(21.0)	10(9.5)	16(17.4)	44(23.7)	12(30.8)	26(20.0)	21(10.0)
Uses CMs**		p<0.001			p 0.394		
No	175(40.7)	49(37.7)	75(58.1)	86(42.2)	24(52.2)	61(39.4)	128(45.2)
Yes	254(59.3)	81(62.3)	54(41.9)	118(57.8)	22(47.8)	94(60.6)	155(54.8)
Total	429(100)	130(100)	129(100)	204(100)	46(100)	155(100)	283(100)

*Indiff. = Indifferent; ^†‡^some women did not answer; ^§^LMUP = London Measure of Unplanned Pregnancy used only for women who already had children; ^‖^Ambiv. = Ambivalent; ^¶^N. pl. = Not planned; **CMs = Contraceptive methods

According to Table 4, women who had a strong desire to avoid pregnancy were, in greater proportion, aged 35 years old and over (p=0.015); had two or more children (p<0.001), and their last pregnancy was not planned (p=0.002). The multiple logistic regression analysis showed that being in a stable union (OR=0.49; 95% CI: 0.33-0.74), having two or more children (OR=15.9; 95% CI: 4.29-59,1), and the last pregnancy not planned (OR=2.94; 95% CI: 1.30-3.83) were associated with a strong desire to avoid pregnancy.

**Table 4:** Number and proportion of women with a strong desire to avoid pregnancy. São Paulo, SP, Brazil, 2018

Variable	Strong desire to avoid pregnancy		Wants to get pregnant/ is ambivalent			OR	95% CI
	n	%	N	%	P		
Age (years old)							
18-24	71	33.2	143	66.8	0.015	1	-
25-34	108	42.3	147	57.6		0.98	0.62-1.54
35+	102	46.6	117	53.4		1.12	0.68-1.86
Schooling							
Elementary	11	52.3	10	47.6	0.067	1	-
High School	241	42.1	332	57.9		0.66	0.24-1.77
Higher	29	30.8	68	59.1		0.51	0.16-1.57
Socioeconomic status							
A+B	60	38.0	98	62.0	0.643	1.0	-
C	201	42.0	278	58.0		0.87	0.56-1.35
D+E	17	43.6	22	56.4		0.63	0.27-1.46
Declared skin color							
White	63	65.2	116	64.8	0.071	1	-
Non-white	218	42.9	290	57.1		1.18	0.80-1.76
Religion							
None	72	40.9	104	59.1	0.675	1	-
Catholic	77	44.0	98	56.0		1.19	0.73-1.94
Evangelical	119	38.6	189	61.4		0.90	0.59-1.37
Others	13	44.8	16	55.2		2.21	0.88-5.54
Own income							
No	146	40.0	219	60.0	0.608	1	-
Yes	135	41.9	187	58.1		1.01	0.71-1.44
Health insurance							
No	238	42.1	327	57.9	0.143	1	-
Yes	43	35.0	80	65.0		1.01	0.63-1.61
Has a partner							
No	102	41.8	142	58.2	0.704		-
Yes	179	40.3	265	59.7		0.49	0.33-0.74
Parity							
None	16	12.5	112	87.5	<0.001	1	-
1 child	95	36.1	168	63.9		6.61	1.97-24.30
2+ children	170	57.2	127	42.8		15.9	4.29-59.10
LMUP*							
Nev. preg.^†^	79	32.6	23	10.2		0.2	0.10-0.50
Planned	77	38.1	125	61.9	0.002	0.69	0.73-0.94
N. pl.^‡^	124	47.9	135	52.1		1.71	0.95-2.14
Ambivalent	61	59.2	42	40.8		2.94	1.30-3.83
Type of CM^§^ used (effectiveness)							
Does not use	182	44.7	007	41.6	0.845	1	-
Low	117	41.6	182	44.7		1.12	0.55-2.25
Mean	1	6.8	26	6.4		1.33	0.91-1.92
Discharge	137	48.7	190	46.7		1.23	0.40-3.71

* LMUP = London Measure of Unplanned Pregnancy;

† Nev. preg. = Never got pregnant;

‡ N. pl. = Not planned;

§ CM = Contraceptive method

There was no statistical difference between having a strong desire to avoid pregnancy and the type of contraceptive method used, i.e., women with a strong desire to avoid pregnancy used basically the same types of contraceptive methods as women in general. Contraceptive use was defined as the use of any contraceptive method during the period of interview. No particular contraceptive method was associated with a strong desire to prevent pregnancy. It is noteworthy that even not using any method was similar (Table 5).

**Table 5:** Number and proportion of women with a strong desire to avoid pregnancy, associated with the use of contraception. São Paulo, SP, Brazil, 2018

Contraceptive method in use	Strong desire to avoid pregnancy				
	No		Yes		p
	n	%	n	%	
None	182	44.7	117	41.6	0.423
Quarterly injection	97	43.5	84	51.2	0.128
Pill	94	42.3	53	32.7	0.055
Male condom	40	18.1	25	15.4	0.492
IUD	10	4.5	8	4.9	0.858
Female condom	1	0.4	1	0.6	0.822
Withdrawal method	1	0.5	1	0.6	0.825
Calendar	1	0.4	1	0.6	0.823

## Discussion

Our study considered feelings, intentions, and attitudes towards a possible future pregnancy with women who had an active sex life, but who did not necessarily have children, aged between 18 and 49 years old, not lacquered, non-vasectomized partners, with a strong desire to avoid pregnancy. Although the majority of participants had a strong desire to avoid pregnancy, their use of contraception was similar to those who did not have this desire. They were ambivalent about pregnancy planning and were using contraceptive methods of medium and low effectiveness.

Even though the non-use of contraceptive methods by a considerable number of women who did not wish to become pregnant can be compared to data from the PNDS, the use of contraceptive methods increased substantially in Brazil, but it cannot be ignored that contraceptive practice is based on subjectivity and not in rationality^(^ 9 ^)^. Although the use of contraceptive methods is high in the country, a nationwide study showed that most women did not intend to become pregnant, wanted to wait longer and had no desire to be mothers at any time^(^ 10 ^)^.

In a prospective analysis with Latin women from the United States of America (USA)-Mexico border, it was investigated to what extent the use of contraceptive methods was associated with the desire to prevent pregnancy. Using the National Survey of Family Growth (NSFG), women who replied that they did not want another pregnancy were not using contraceptive methods nor did they care about getting pregnant^(^ 17 ^,^ 20 ^)^.

Pregnancy intentions can be complex, involving a variety of emotional and psychological factors, the product of individual intentions, and multiple intertwined social and economic influences, including community, partner, and personal values about pregnancy. Understanding a woman’s pregnancy intentions can help to ensure that she uses more effective and/or more consistent methods, thereby reducing the likelihood of an unintended pregnancy, provided they have access to the means to do so^(^ 20 ^-^ 21 ^)^.

The relation between motivation to avoid pregnancy and incongruous intentions and feelings is often examined by looking at the type of contraceptive method used and its correct use. There is evidence that women’s ambivalence in avoiding pregnancy is associated with inconsistent or incorrect use of contraceptives or with the use of less effective methods. Thus, the use of contraceptive methods may not occur consistently and continuously, resulting in situations of contraceptive vulnerability^(^ 22 ^)^.

When women express the intention of becoming pregnant, their contraceptive behaviors are not necessarily congruent. Given the emotional, psychological, and cultural factors, behaviors often do not align with intentions as well as intentions can change over time. Many women express ambivalence about their intentions to become pregnant. Formulating plans for a pregnancy may seem unrealistic to many, as they do not perceive themselves as having reproductive control^(^ 22 ^)^.

Another consideration is whether the use of contraceptives alone should be interpreted as evidence of an intention to prevent pregnancy. In this study, most women who were users of some type of contraceptive method answered that it was very important to prevent pregnancy and, if pregnancy occurred, this would be at the wrong time, but they would feel happy, showing ambivalent feelings.

A study carried out in the USA between 2008, 2012 and 2014 on the use of contraceptive methods showed that women used and discontinued the use of methods based on the characteristics of these methods, including side effects, efficacy and ease of use^(^ 17 ^)^ being limited to access, planned services, discrimination in health care environments, and financial barriers^(^ 18 ^)^.

Many women may find methods difficult to use correctly because they are dissatisfied with certain aspects, such as interference with sexual function, negative side effects, or non-acceptance by intimate partners (e.g. male and female condoms and pill)^(^ 23 ^-^ 24 ^)^.

In this regard, The Contraceptive CHOICE Project (CHOICE) sought to reduce unwanted pregnancies by removing barriers to cost, education, and access to highly effective contraceptives. It was a prospective cohort study of more than 9,000 women aged 14 to 45 years old who received staggered contraceptive counseling to raise awareness of all available reversible methods, particularly Long-Acting Reversible Contraceptive (LARC) methods. Most of the study participants chose the levonorgestrel intrauterine device, subdermal implant, and copper intrauterine device respectively^(^ 25 ^)^, generating substantial cost savings due to increased acceptance of highly effective contraceptives and consequent prevention of unwanted pregnancies and births^(^ 26 ^)^.

Our results showed that a considerable proportion of women who had a strong desire to avoid pregnancy did not use contraception methods. For those who used some method, the use of medium and low efficiency methods was verified^(^ 19 ^)^, which shows that women may not be supported to achieve their reproductive preferences.

An analysis of the prevalence of modern and traditional contraceptive methods by type of method in Brazil found that most women used the pill or did not use any method^(^ 23 ^)^, corroborating our results.

It is imperative that the health services organize themselves to offer quality and quantity contraceptives to meet the demands of the users. The lack of contraceptives or even the lack of access and supply are among the most cited reasons in low- and middle- income countries for unmet demand, non-use, and discontinuation of contraception^(^ 23 ^)^.

Contraceptive availability goes beyond simply supporting better health for women. It is important to develop and establish reliable systems in the supply chain to ensure that goods and services meet women’s contraceptive needs. If efficient, they improve the quality of care and support for choosing modern methods of contraception. Strengthening the supply chain can improve contraceptive security, as all customers will be able to freely choose, obtain and use good quality contraceptives^(^ 27 ^)^.

The limiting aspect of this investigation is its execution limited to one region and the non-inclusion of all regions of the city. Thus, its replication is recommended to learn about other scenarios. Despite this limitation, the results of the present study may bring new contributions to elucidate the intention to become pregnant, the importance of preventing it, the opportune moment to get pregnant, feeling about unexpected pregnancy, not using contraceptive methods or discontinuity associated with the intentionality of the pregnancy.

## Conclusion

Assessing pregnancy intention is an essential element to understand why women with a strong desire to avoid pregnancy use the same types of contraceptive methods as women in general. This study confirms the strong relation between unintended pregnancy, ambivalence, and the use or not of contraceptive methods, indicating the need for public policies that guarantee not only access, but the expansion of options for more effective contraceptive methods. The evidence suggests a promising path for future research on the health impacts of unintended pregnancy.
